# Precision oncology in the treatment of patients with bone sarcomas: an up-to-date narrative review

**DOI:** 10.37349/etat.2026.1002396

**Published:** 2026-08-09

**Authors:** Sude Yarar, Panagiotis Tsagkozis

**Affiliations:** IRCCS Azienda Ospedaliero-Universitaria di Bologna, Italy; ^1^Istanbul Faculty of Medicine, Istanbul University, Istanbul 34452, Turkey; ^2^Department of Molecular Medicine and Surgery, Karolinska Institutet, 171 76 Stockholm, Sweden; ^3^Karolinska University Hospital, 171 64 Solna, Sweden

**Keywords:** bone sarcoma, functional assays, drug sensitivity, precision oncology

## Abstract

Primary bone sarcomas are rare and biologically heterogeneous malignancies for which therapeutic progress remains limited, particularly in metastatic and recurrent disease. Advances in genomic and molecular profiling have revealed substantial inter- and intratumoral heterogeneity across the major subtypes, including osteosarcoma, Ewing sarcoma and chondrosarcoma, challenging conventional histology-driven treatment strategies. Precision medicine approaches are being increasingly explored to better capture this biological complexity and guide individualized therapeutic decision-making. This review examines emerging precision oncology strategies in bone sarcomas, including molecular diagnostics, targeted therapeutic approaches, three-dimensional functional modeling systems, and liquid biopsy technologies for dynamic disease monitoring. Together, these platforms provide biologically informed frameworks for patient-specific treatment and longitudinal assessment of tumor evolution. However, clinical implementation remains limited by genomic complexity, small patient cohorts, and methodological variability across experimental platforms. The integration of multi-layered precision models combining genomic stratification, functional drug sensitivity testing and circulating biomarker monitoring may enable more adaptive and individualized management strategies. Such approaches have the potential to improve therapeutic selection and ultimately advance outcomes for patients with primary bone sarcomas.

## Introductıon

Bone sarcomas are rare and heterogeneous malignant mesenchymal tumors arising from osseous tissue, accounting for approximately 0.2% of all malignant tumors [1]. Despite their low incidence, these tumors represent a major clinical challenge due to their aggressive biological behavior, early metastatic potential, and disproportionate impact on children, adolescents, and young adults [2]. Osteosarcoma, Ewing sarcoma, and chondrosarcoma constitute the most common primary bone sarcoma subtypes, each characterized by distinct epidemiological patterns, molecular drivers, and clinical trajectories [1].

Historically, bone sarcomas have been classified and managed primarily according to histopathological features and anatomical location. However, accumulating genomic and epigenetic evidence demonstrates that these tumors comprise biologically stratified entities rather than a single disease spectrum [3]. Large-scale clinical genomic profiling studies have revealed subtype-specific alterations, recurrent gene rearrangements, and potentially actionable molecular events across sarcoma subtypes [4]. In parallel, DNA methylation-based classification and other molecular diagnostic tools have improved tumor categorization in cases where morphology alone is insufficient [5]. These findings underscore the profound inter- and intratumoral heterogeneity that underlies clinical variability and therapeutic resistance.

Despite advances in molecular characterization, therapeutic progress has been comparatively modest. Multimodal treatment combining surgery, chemotherapy, and radiotherapy has improved outcomes in localized disease, yet survival rates for metastatic, recurrent, or refractory bone sarcomas remain unsatisfactory [6]. Moreover, current treatment paradigms largely rely on standardized regimens that do not systematically incorporate molecular stratification or predictive biomarkers.

In parallel with advances in molecular profiling, digital health technologies and Internet of Things (IoT)-based systems are increasingly being integrated into oncologic practice. In musculoskeletal oncology, IoT-enabled platforms may support real-time imaging integration, surgical navigation, wearable postoperative monitoring, and remote multidisciplinary collaboration, potentially contributing to more individualized management strategies for patients with bone sarcomas [7].

The expanding understanding of bone sarcoma biology has therefore increased interest in precision medicine approaches aimed at integrating genomic profiling, dynamic disease monitoring, and patient-specific therapeutic strategies.

## Current treatment of bone sarcomas

Current management of bone sarcomas relies on multimodal treatment integrating surgery, systemic chemotherapy, and radiotherapy. Despite subtype-specific differences, therapeutic strategies remain primarily guided by histopathological classification rather than molecular stratification [8].

Surgical resection with negative margins represents the cornerstone of curative treatment for localized bone sarcomas [9]. Limb-sparing procedures are now feasible in most patients, although metastatic burden, tumor biology, and resectability continue to influence long-term outcomes [10, 11]. Radiotherapy is mainly incorporated in Ewing sarcoma because of its relative radiosensitivity, whereas osteosarcoma and conventional chondrosarcoma demonstrate relative radioresistance [12]. Systemic multi-agent chemotherapy constitutes a central component of treatment for osteosarcoma and Ewing sarcoma, whereas its role in conventional chondrosarcoma remains limited, as summarized in Table 1 [13].

**Table 1:** Current standard treatment strategies and major limitations across primary bone sarcoma subtypes.

**Subtype**	**Surgery**	**Chemotherapy regimen**	**Radiotherapy**	**Major limitations**
Osteosarcoma	Wide resection, limb-sparing	MAP (HD-MTX, doxorubicin, cisplatin) ± ifosfamide	Limited role	Plateau in survival, chemoresistance
Ewing sarcoma	Surgery ± RT	VDC/IE	Frequently used	Poor metastatic outcomes
Chondrosarcoma	Primary treatment	Limited efficacy	Limited role	Chemoresistance

MAP: methotrexate–doxorubicin–cisplatin; HD-MTX: high-dose methotrexate; VDC/IE: vincristine–doxorubicin–cyclophosphamide/ifosfamide–etoposide; RT: radiotherapy.

In osteosarcoma, the introduction of methotrexate–doxorubicin–cisplatin (MAP)-based chemotherapy (high-dose methotrexate, doxorubicin, and cisplatin) significantly improved survival in localized disease; however, outcomes for metastatic or recurrent disease have remained largely unchanged over recent decades [14]. Attempts to intensify postoperative regimens have not consistently improved survival outcomes [15]. Current osteosarcoma treatment strategies remain largely standardized despite increasing evidence that genomic variability influences therapeutic response and progression-free survival [16]. Ewing sarcoma is commonly treated with dose-intensive vincristine–doxorubicin–cyclophosphamide/ifosfamide–etoposide (VDC/IE)-based chemotherapy combined with surgery and/or radiotherapy, achieving improved outcomes in localized disease but limited survival in relapsed or metastatic settings [17]. Conventional chondrosarcoma demonstrates intrinsic resistance to cytotoxic chemotherapy, leaving surgery as the primary treatment modality in most patients [18].

## Other oncological treatments

The limited improvement in outcomes for metastatic and recurrent primary bone sarcomas has accelerated interest in targeted therapies as a core pillar of precision medicine. However, unlike many epithelial cancers in which high-frequency, druggable driver mutations enable relatively direct biomarker-to-therapy matching, bone sarcomas are frequently characterized either by complex genomic instability (osteosarcoma), fusion-driven transcriptional programs with low mutational burden (Ewing sarcoma), or subtype-dependent metabolic/epigenetic alterations with limited systemic options (chondrosarcoma). Consequently, targeted treatment development has largely centered on pathway-level dependencies, tumor-microenvironment signaling, and rational combinations rather than single-driver inhibition, and the clinical impact of targeted agents has been variable and often modest [19, 20].

In osteosarcoma, the absence of a dominant recurrent oncogenic driver has shifted therapeutic development toward targeting multi-kinase signaling, angiogenesis, and downstream growth pathways [21]. Clinically, multi-kinase inhibitors and pathway-directed agents have been investigated as strategies to suppress proliferative and pro-metastatic signaling in this genomically unstable disease [22]. Patient-derived xenograft (PDX) models further illustrate the translational rationale for these approaches, identifying regimens with activity in drug-resistant osteosarcoma, including regorafenib monotherapy, irinotecan-based combinations, and multi-agent strategies incorporating kinase and mTOR-pathway inhibition [23, 24]. These observations highlight a central principle of precision medicine in osteosarcoma: therapeutic vulnerabilities often emerge from pathway-level network dependencies rather than from single genomic alterations. This may explain why apparent “actionable” alterations identified through molecular profiling do not consistently translate into durable clinical benefit without complementary functional validation.

Ewing sarcoma provides a distinct paradigm for targeted therapy development. Although it is defined by EWSR1–ETS (EWS RNA-binding protein 1–ETS transcription factor fusion family) fusions, the disease-defining transcription factor remains difficult to target directly, and the overall scarcity of additional recurrent mutations limits classical mutation-driven precision oncology [25, 26]. As a result, translational efforts have focused on downstream signaling and epigenetic maintenance of the fusion-driven state. Ewing-targeted strategies have focused on IGF1R signaling, although the field has been constrained by the lack of currently recruiting trials and by the need for predictive biomarkers to identify responders. In parallel, developmental pathways and epigenetic modulators have emerged as major targets given the centrality of epigenetic dysregulation in fusion-driven sarcomas. Preclinical and early clinical efforts have explored HDAC inhibition, EZH2 targeting, BET inhibition, and LSD1 inhibition; notably, LSD1 inhibitors have progressed into phase I clinical evaluation, and preclinical data suggest that combinatorial epigenetic targeting may enhance activity compared with monotherapy [27–29]. Beyond epigenetic drugs, surface targets such as CD99 have been proposed as antibody-directed approaches, illustrating an alternative precision logic based on lineage-associated cell-surface dependencies rather than mutational drivers [25, 30]. Together, these strategies exemplify how precision medicine in Ewing sarcoma often relies on identifying pathways that sustain fusion-mediated transcription or mediate microenvironmental crosstalk, rather than targeting a high-frequency kinase mutation.

Chondrosarcoma remains one of the most challenging major bone sarcoma subtypes from a systemic therapy perspective, largely due to its intrinsic resistance to conventional cytotoxic chemotherapy and, in many cases, limited sensitivity to radiotherapy [31]. These features have motivated the development of molecularly informed strategies focused on recurrent metabolic and developmental pathway alterations. Mutations in IDH1 and IDH2 represent a central molecular axis in many chondrosarcomas, providing a biologically coherent rationale for mutant-IDH inhibition as a targeted therapeutic strategy [32, 33]. In addition, several signaling pathways have been proposed as potential therapeutic targets, including Hedgehog signaling, mTOR signaling, SRC/AKT pathway activity, and receptor tyrosine kinase alterations such as IGF1R and KIT amplification in specific subsets [18, 34]. While these approaches represent conceptually strong precision-medicine strategies, clinical benefit has frequently been limited or heterogeneous. This observation aligns with the broader view that chondrosarcoma comprises biologically diverse entities in which molecular stratification may be necessary but not sufficient to ensure therapeutic response.

### Tyrosine kinase inhibitors (TKIs) and anti-angiogenic strategies

TKIs represent one of the most clinically advanced targeted therapy approaches in recurrent and unresectable bone sarcomas. Their rationale is based on the involvement of multiple receptor tyrosine kinases, including VEGFR, PDGFR, FGFR, KIT, RET, MET, AXL, and IGF1R, in angiogenesis, tumor proliferation, metastatic progression, and microenvironmental signaling. Because osteosarcoma and Ewing sarcoma rarely depend on a single dominant druggable driver, newer multi-receptor TKIs may be more clinically relevant than highly selective single-target agents. Recent reviews emphasize that anti-angiogenic multi-RTK inhibitors combine VEGFR blockade with simultaneous inhibition of additional pathways such as PDGFR, FGFR, KIT, RET, MET, or AXL, providing a rational strategy for biologically heterogeneous tumors [35].

In osteosarcoma, the strongest clinical evidence has emerged for regorafenib and cabozantinib. Two randomized phase II studies showed that regorafenib improved progression-free survival in recurrent or metastatic osteosarcoma compared with placebo, although no clear overall survival benefit was demonstrated [36, 37]. Cabozantinib has also shown encouraging activity in heavily pretreated osteosarcoma, with reported partial responses and disease control in a clinically meaningful subset of patients [38]. Other anti-angiogenic TKIs, including sorafenib, apatinib, anlotinib, lenvatinib, and pazopanib, have demonstrated varying degrees of disease stabilization or response in phase II, retrospective, or early clinical studies.

Despite these advances, the benefit of TKIs in osteosarcoma remains limited by acquired resistance, modest durability of response, toxicity, and the lack of validated predictive biomarkers. This explains why most studies show improvements in progression-free survival rather than overall survival [39]. Current evidence therefore suggests that TKIs are unlikely to serve as durable single-agent solutions in osteosarcoma. Instead, their future role may depend on biomarker-guided patient selection and rational combinations with chemotherapy, mTOR or MEK pathway inhibition, and immune checkpoint blockade [40, 41].

In Ewing sarcoma, the evidence base is less mature, but regorafenib and cabozantinib have demonstrated antitumor activity in phase II settings [38, 42, 43]. Cabozantinib produced objective responses and disease control in both Ewing sarcoma and osteosarcoma cohorts, while regorafenib also showed clinical activity in advanced Ewing sarcoma [38]. Anlotinib combined with chemotherapy has shown particularly notable response rates in early studies, although randomized comparisons are still needed to define the independent contribution of the TKI component [44, 45].

Overall, anti-angiogenic multi-RTK inhibitors represent one of the most promising therapeutic directions in advanced bone sarcomas. However, their current clinical value lies mainly in temporary disease control rather than durable remission. Future trials should prioritize molecular response biomarkers, earlier integration in selected patients, rational combination strategies, and toxicity-conscious dosing schedules to improve the therapeutic index of this drug class.

### PARP inhibition and DNA damage response-targeted therapies

Poly(ADP-ribose) polymerase (PARP) inhibition has emerged as a promising DNA damage response-targeted therapeutic strategy in primary bone sarcomas, particularly osteosarcoma and Ewing sarcoma. Interest in this approach has been driven by increasing recognition of homologous recombination deficiency, genomic instability, and defective DNA repair signaling within these tumors. In osteosarcoma, genomic and epigenomic analyses have identified mutational signatures and chromosomal instability patterns resembling BRCA-deficient malignancies, leading to the concept of “BRCAness” in subsets of osteosarcoma [46]. These findings provide a biological rationale for exploiting synthetic lethality through PARP inhibition.

Despite this rationale, the therapeutic role of PARP inhibitors in osteosarcoma remains incompletely defined. Preclinical studies demonstrated that osteosarcoma cell lines with homologous recombination deficiency-associated molecular features may exhibit sensitivity to PARP inhibition, particularly when combined with DNA-damaging chemotherapy. Talazoparib combined with temozolomide showed synergistic induction of apoptosis and suppression of clonogenic survival in BRCAness-associated osteosarcoma models [47]. Similarly, olaparib combined with doxorubicin enhanced antitumor activity in vitro and in vivo, supporting the concept that PARP inhibition may potentiate chemotherapy-induced DNA damage in osteosarcoma [48]. However, broader chemosensitivity analyses have demonstrated substantial biological heterogeneity among osteosarcoma models, with inconsistent PARP inhibitor responsiveness despite genomic features suggestive of BRCAness [49]. Consequently, although PARP inhibition represents a biologically compelling strategy, its clinical application in osteosarcoma currently remains investigational, and validated predictive biomarkers are still lacking.

In Ewing sarcoma, the rationale for PARP inhibition appears stronger because of the direct interaction between EWS–FLI1 fusion signaling and PARP1-mediated DNA repair pathways. Early mechanistic studies demonstrated that EWS fusion proteins interact with PARP1 and promote dependence on PARP-associated transcriptional and DNA damage response mechanisms [50]. Subsequent translational studies further showed that PARP inhibition may enhance sensitivity to chemotherapy and radiotherapy through amplification of DNA damage and impaired repair capacity. Combination approaches involving PARP inhibitors with trabectedin, temozolomide, irinotecan, or radiation therapy have demonstrated synergistic antitumor activity in preclinical Ewing sarcoma models and PDXs [51, 52]. Recent work combining talazoparib, irinotecan, and radiation therapy further demonstrated prolonged survival and reduced tumor burden in orthotopic Ewing sarcoma xenograft models, supporting the potential role of PARP-based radiosensitization strategies in refractory disease settings [52].

Although these findings position PARP inhibition among the most biologically promising precision oncology strategies in bone sarcomas, several challenges remain. Clinical responses have been variable, acquired resistance mechanisms are incompletely understood, and durable benefit in prospective clinical trials has not yet been firmly established. Current research therefore increasingly focuses on biomarker-guided patient selection, identification of homologous recombination deficiency signatures, and rational combination strategies integrating PARP inhibitors with chemotherapy, radiotherapy, or other targeted agents.

## Limitations of the current paradigm

Despite aggressive multimodal therapy, outcomes for metastatic, recurrent, and treatment-resistant bone sarcomas remain poor. Current treatment strategies are largely histology-driven and fail to adequately account for the substantial genomic and molecular heterogeneity observed across bone sarcoma subtypes [53]. In addition, chemoresistance, cumulative toxicity, and limited ability to predict therapeutic response continue to restrict clinical progress.

These limitations underscore the need for precision oncology approaches integrating molecular profiling, dynamic disease monitoring, and patient-specific therapeutic selection into routine clinical practice [54].

## Precision medicine strategies in bone sarcomas

### Biological and molecular landscape of bone sarcomas

Bone sarcomas are biologically heterogeneous malignancies characterized by substantial genomic and molecular diversity. Large-scale profiling studies have identified subtype-specific alterations across osteosarcoma, Ewing sarcoma, and chondrosarcoma, demonstrating that histopathological classification alone insufficiently captures tumor biology and therapeutic vulnerability [55]. This molecular heterogeneity contributes to treatment resistance, disease progression, and variable clinical outcomes, while also providing the biological rationale for precision oncology approaches in bone sarcomas [56].

Bone sarcoma subtypes exhibit distinct genomic architectures that influence therapeutic strategies. Osteosarcoma is characterized by extensive chromosomal instability and widespread structural alterations involving pathways such as TP53 and RB1, contributing to marked intratumoral heterogeneity and variable treatment response [57, 58]. In contrast, Ewing sarcoma is primarily driven by recurrent EWSR1–ETS fusion proteins, most commonly EWSR1–FLI1, which promote oncogenesis through transcriptional and epigenetic dysregulation despite a relatively low mutational burden [59, 60]. Chondrosarcoma frequently harbors IDH1 and IDH2 mutations associated with metabolic and epigenetic alterations, although biological behavior varies substantially across subtypes [61].

Collectively, these findings highlight that bone sarcomas represent molecularly distinct diseases rather than a single clinical entity, supporting the development of biologically informed and subtype-specific therapeutic strategies.

#### Molecular diagnostics and genomic stratification

Molecular profiling has increasingly redefined primary bone sarcomas as biologically heterogeneous diseases rather than a single histology-driven spectrum. Clinically deployed next-generation sequencing (NGS) can redefine diagnosis, identify subtype-defining alterations, and detect potentially actionable molecular events, supporting its role as a central component of precision oncology [4, 62]. However, clinically actionable mutations remain relatively uncommon in many bone sarcomas, and the therapeutic relevance of genomic findings is often context-dependent, requiring complementary functional validation [63].

**Figure 1:**
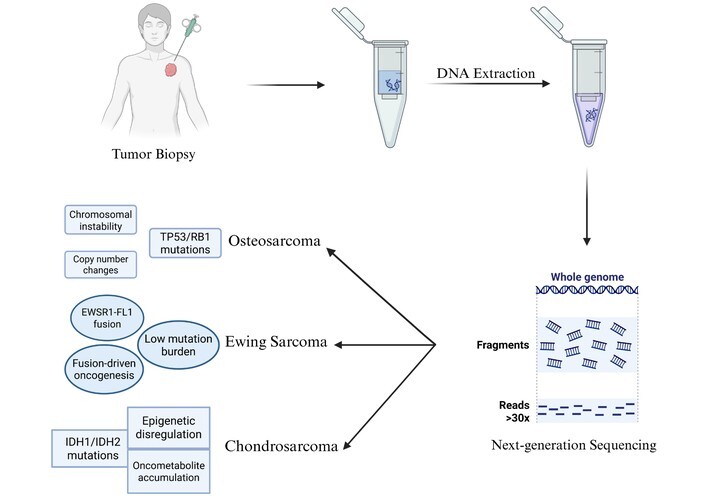
**Molecular profiling workflow and representative genomic alterations in major bone sarcoma subtypes.** Created in BioRender. Tsagkozis, P. (2026) https://BioRender.com/02lutz3.

The precision oncology landscape differs substantially across bone sarcoma subtypes, as shown in Figure 1 and Table 2. Osteosarcoma is characterized by complex genomic architecture with extensive chromosomal instability, structural rearrangements, and copy-number alterations rather than recurrent targetable driver mutations [64, 65]. This complexity contributes to marked intratumoral heterogeneity and variable therapeutic response, limiting the effectiveness of single-target strategies [66]. Comprehensive profiling studies have identified multiple pathway-level abnormalities in refractory osteosarcoma; however, matched targeted therapies have frequently produced limited clinical benefit, highlighting the gap between molecular actionability and therapeutic efficacy [67]. Emerging approaches therefore increasingly focus on pathway-level dependencies, resistance biology, and regulatory signaling networks rather than isolated genomic alterations [68, 69]. Pharmacogenomic variability and regulatory mechanisms such as non-coding RNAs have also been implicated in treatment response and therapeutic resistance, particularly through pathways including Wnt/β-catenin signaling [70, 71].

**Table 2:** Molecular characteristics and precision oncology implications across major bone sarcoma subtypes.

**Subtype**	**Dominant genomic architecture**	**Highest-yield profiling targets**	**Primary precision output**	**Main limitation**
Osteosarcoma	Complex-karyotype; chromosomal instability	Copy-number alterations, structural variants, pathway-state inference	Biological risk stratification; candidate targets often pathway-level	Actionability is diffuse; matched therapy benefit inconsistent
Ewing sarcoma	Fusion-driven (EWSR1–ETS); low mutation burden	Fusion identification/breakpoint assays; recurrent events (e.g., STAG2/CDKN2A)	Diagnostic confirmation/refinement; monitoring-amenable biomarkers	Few recurrent druggable point mutations; biomarker validation variable
Chondrosarcoma	Subtype-dependent; metabolic/epigenetic axis common	IDH1/2 status; genomic instability signatures (e.g., LOH burden); transcriptomic state	Molecular subgrouping beyond grade; trial stratification	Clinical translation of subgrouping still limited

EWSR1–ETS: EWS RNA-binding protein 1–ETS transcription factor fusion family; STAG2: stromal antigen 2; CDKN2A: cyclin dependent kinase inhibitor 2A; IDH1/2: isocitrate dehydrogenase 1 and 2; LOH: loss of heterozygosity.

In contrast, Ewing sarcoma is a fusion-driven malignancy primarily defined by EWSR1–ETS rearrangements, most commonly EWSR1–FLI1 [59]. Compared with osteosarcoma, Ewing sarcoma exhibits a relatively low mutational burden, shifting precision strategies toward fusion characterization and downstream signaling dependencies rather than mutation-based targeting [72, 73]. Molecular profiling additionally improves diagnostic accuracy and clinical trial stratification by identifying alternative fusion subtypes and biologically distinct transcriptional programs [74, 75]. However, many proposed biomarkers remain inconsistently validated across cohorts, highlighting the need for prospective validation before routine clinical implementation [76]. Recent studies further suggest that microenvironmental signaling networks and receptor-kinase pathways, including VEGFR2, MET, and AXL, may represent therapeutically relevant targets in Ewing sarcoma [77–79].

Chondrosarcoma demonstrates a distinct molecular landscape frequently associated with IDH1 and IDH2 mutations and epigenetic dysregulation [80]. However, emerging genomic and transcriptomic studies indicate that IDH status alone does not fully capture the biological heterogeneity of the disease [81–83]. Integrated molecular stratification approaches incorporating genomic instability patterns and expression-defined cellular states may therefore provide improved biological classification and future therapeutic guidance.

Collectively, molecular diagnostics and genomic stratification provide an essential foundation for precision medicine in bone sarcomas, although genomic profiling alone is often insufficient to predict therapeutic response. Increasingly, integrated precision oncology frameworks aim to combine genomic characterization with functional validation, molecular monitoring, and subtype-specific therapeutic targeting.

#### Targeted therapies

##### Adoptive cellular treatment

Adoptive cellular therapies are emerging as an important next-generation precision oncology strategy in bone sarcomas, particularly for relapsed or treatment-refractory disease. These approaches include chimeric antigen receptor T-cell (CAR-T) therapy, T-cell receptor-engineered therapies (TCR-T), and NK-cell-based immunotherapy. Unlike immune checkpoint blockade, adoptive cellular therapies directly redirect immune effector cells toward tumor-associated antigens and may therefore overcome some of the immune resistance mechanisms observed in bone sarcomas [84].

In osteosarcoma, several tumor-associated antigens have been investigated as potential CAR-T targets, including B7-H3, GD2, HER2, and LRRC15 [85]. Among these, B7-H3 has emerged as one of the most promising candidates because of its frequent overexpression and association with metastatic progression and poor prognosis. Preclinical studies demonstrated significant antitumor activity of B7-H3-directed CAR-T cells in osteosarcoma models, including pulmonary metastatic disease. HER2-directed CAR-T therapy has likewise shown early clinical feasibility in sarcomas, although therapeutic responses remain heterogeneous [86, 87]. More recently, LRRC15-targeted CAR-T cells demonstrated encouraging preclinical antitumor activity in osteosarcoma models, supporting continued investigation of stromal-associated therapeutic targets [88].

TCR-engineered therapies are also being explored in sarcomas, particularly against cancer-testis antigens such as NY-ESO-1, MAGE-family antigens, and PRAME. Early clinical studies in synovial sarcoma demonstrated therapeutic activity of NY-ESO-1-directed TCR-engineered lymphocytes, supporting the broader rationale for adoptive TCR-based therapies in mesenchymal malignancies. Similar approaches are currently under investigation in osteosarcoma and Ewing sarcoma, although clinical data remain limited [89]. Compared with CAR-T approaches, TCR-engineered therapies may provide advantages in tumors with heterogeneous or low surface-antigen expression because they recognize intracellular antigens presented through HLA molecules.

In Ewing sarcoma, GD2- and VEGFR2-directed CAR-T strategies have demonstrated preclinical antitumor activity, particularly when combined with approaches targeting the immunosuppressive tumor microenvironment [90, 91]. Cellular therapy development in chondrosarcoma remains less advanced; however, recent studies suggest that B7-H3-directed CAR-T cells and epigenetic sensitization strategies may represent promising future therapeutic approaches, particularly in dedifferentiated subtypes resistant to conventional therapies [92].

Beyond CAR-T and TCR-T therapies, NK-cell-based adoptive immunotherapy is also being explored in pediatric and adolescent bone sarcomas because of its potential for MHC-independent tumor killing and lower risk of graft-versus-host disease [89]. Nevertheless, despite encouraging translational findings, most adoptive cellular therapy approaches in bone sarcomas remain in preclinical or early-phase clinical development. Current research increasingly focuses on biomarker-guided target selection, improvement of immune-cell trafficking and persistence, and rational combination strategies designed to overcome the immunosuppressive tumor microenvironment.

##### Immunotherapy

In parallel with subtype-specific pathway targeting, immune-based strategies are increasingly incorporated into precision oncology frameworks for bone sarcomas. Several studies have shown that bone sarcomas express immune checkpoint molecules such as PD-L1, PD-L2, and B7-H3, providing a biological rationale for checkpoint blockade and immune-directed clinical trials [93, 94]. However, review literature from the immunotherapy era emphasizes that bone sarcomas frequently exhibit immunosuppressive tumor microenvironments and heterogeneous checkpoint expression patterns across subtypes. These features likely contribute to inconsistent clinical responses and highlight the need for biomarker-guided patient selection and rational combination approaches. In this context, immunotherapy in bone sarcomas represents a precision challenge analogous to kinase targeting: the key question is not simply whether a target exists, but whether the tumor–host immune context is permissive for therapeutic response [95, 96].

Clinical responses to immune checkpoint inhibitors in bone sarcomas have generally remained limited. Early-phase studies investigating PD-1 and PD-L1 inhibitors, including pembrolizumab and nivolumab, demonstrated relatively low objective response rates in unselected osteosarcoma and Ewing sarcoma populations [71, 72]. These findings likely reflect the low tumor mutational burden, heterogeneous immune infiltration, and highly immunosuppressive tumor microenvironment characteristic of many bone sarcomas [69, 70]. Consequently, current research increasingly focuses on combination strategies integrating checkpoint blockade with anti-angiogenic therapies, radiotherapy, chemotherapy, or adoptive cellular therapies in an effort to enhance immune activation and improve therapeutic responsiveness [95].

Collectively, targeted therapies in bone sarcomas illustrate both the promise and the constraints of precision medicine in rare, heterogeneous mesenchymal malignancies. Across subtypes, therapeutic benefit appears highly context-dependent and often requires multi-layer molecular stratification—including genomic architecture, pathway state, and microenvironmental signaling [97]. Increasingly, functional validation strategies are also being incorporated to identify patients most likely to benefit from targeted interventions. These challenges provide a strong rationale for integrating targeted therapy development with dynamic monitoring approaches such as liquid biopsy and with patient-derived functional platforms, including organoids and PDOX models, which may help bridge the persistent gap between molecular “actionability” and clinically meaningful clinical response.

##### Metabolic targeting

Metabolic reprogramming has emerged as an important therapeutic vulnerability in chondrosarcoma, particularly in high-grade and dedifferentiated subtypes that remain largely resistant to conventional chemotherapy and radiotherapy [98]. Among the most clinically relevant alterations are mutations in isocitrate dehydrogenase 1 and 2 (IDH1/2), which are frequently identified in conventional and dedifferentiated chondrosarcomas and contribute to tumor progression through production of the oncometabolite *D*-2-hydroxyglutarate (2-HG) [99, 100].

These findings provided the rationale for the development of IDH-targeted therapies in chondrosarcoma. In a phase I study involving patients with advanced IDH1-mutant chondrosarcoma, the IDH1 inhibitor ivosidenib demonstrated a favorable safety profile and prolonged disease stabilization in a subset of patients, supporting the feasibility of metabolism-directed precision therapy in molecularly selected populations [101]. Although objective responses remain limited, these early clinical findings highlight the therapeutic relevance of metabolic targeting strategies in chondrosarcoma.

Additional metabolic pathways including PI3K-AKT-mTOR signaling, hypoxia-associated pathways, and glutamine metabolism have also emerged as potential therapeutic targets [102]. Experimental studies demonstrated that mTOR inhibition suppresses metabolic activity and tumor growth in chondrosarcoma models, supporting mTOR signaling as a potential pro-survival metabolic pathway [103]. Consequently, current research increasingly focuses on combination approaches integrating metabolic inhibition with epigenetic therapies, anti-angiogenic agents, or immunotherapy.

Overall, metabolic targeting represents one of the most promising emerging precision oncology strategies for advanced chondrosarcoma. However, most approaches remain at the preclinical or early translational stage, and further studies are required to identify predictive biomarkers and optimize combination-based therapeutic strategies.

#### Three-dimensional (3D) tumor models in precision medicine for bone sarcomas

##### Conceptual rationale for 3D modeling

Bone sarcomas, including osteosarcoma, Ewing sarcoma, and chondrosarcoma, exhibit substantial genomic heterogeneity and complex interactions with the bone microenvironment, contributing to therapeutic resistance and variable clinical outcomes [56]. Although advances in molecular profiling have improved biological understanding, genotype-based stratification alone has not consistently translated into clinically actionable precision strategies, particularly in metastatic and recurrent disease.

One limitation of current precision approaches lies in the experimental systems used to evaluate therapeutic response. Conventional two-dimensional (2D) cultures inadequately reproduce the spatial organization and microenvironmental complexity of bone sarcomas, often limiting the predictive accuracy of preclinical drug testing [104]. Consequently, 3D tumor models have emerged as more physiologically relevant platforms capable of better recapitulating tumor heterogeneity, extracellular matrix interactions, and therapy resistance mechanisms [105]. Within a precision oncology framework, these systems may provide functional validation of therapeutic sensitivity beyond molecular profiling alone, and their main characteristics are shown in Table 3.

**Table 3:** Comparison of three-dimensional tumor modeling platforms in bone sarcoma precision medicine.

**Model type**	**Biological fidelity**	**Main precision application**	**Key strength**	**Main limitation**
Spheroids	Moderate (hypoxia and diffusion gradients)	Rapid drug screening	Simple and scalable	Limited matrix and niche realism
Scaffold-based systems	High (bone-mimetic stiffness and structure)	Local therapy + regenerative integration	Microenvironment realism	Manufacturing and clinical translation challenges
PDOs	High (tumor-intrinsic heterogeneity preserved)	Patient-specific drug response testing	Functional therapeutic prediction	Limited immune/stromal representation
PDX/PDOX models	Very high (in vivo architecture)	Regimen validation and resistance modeling	Whole-tumor fidelity	Cost, time, murine stroma
Biofabricated/Chip systems	Emerging high	Spatial and kinetic drug modeling	Architectural precision	Experimental stage

PDOs: patient-derived organoids; PDX: patient-derived xenograft.

##### Multicellular tumor spheroids

Multicellular tumor spheroids represent one of the simplest 3D tumor platforms and more closely reproduce the spatial organization and microenvironmental conditions of bone sarcomas compared with conventional monolayer cultures [106]. In bone sarcoma research, spheroid models have demonstrated increased chemoresistance and improved preservation of extracellular matrix interactions and invasion dynamics, highlighting their value for studying microenvironment-driven therapeutic resistance [107, 108].

From a precision medicine perspective, spheroids provide a relatively scalable and physiologically relevant platforms for preclinical drug testing. However, limitations including incomplete replication of bone-specific mechanical properties and limited stromal or immune representation restrict their ability to fully model the native tumor microenvironment [107, 108].

##### Scaffold-based 3D engineering

Scaffold-based 3D systems use engineered biomaterials to better reproduce the structural and mechanical properties of the bone microenvironment, which plays an important role in bone sarcoma progression [109]. Beyond improving biological realism, these platforms may also support localized drug delivery and regenerative reconstruction strategies [110].

Preclinical studies have demonstrated that scaffold-based systems can simultaneously promote tumor control and bone regeneration, highlighting their potential translational value in osteosarcoma management [111, 112]. However, despite these promising findings, scaffold platforms remain limited by technical complexity, regulatory challenges, and the lack of large-scale clinical validation [113].

##### Patient-derived organoids (PDOs) and PDX models

PDOs have emerged as one of the most clinically relevant 3D tumor platforms in bone sarcoma precision medicine, preserving patient-specific tumor architecture, genomic heterogeneity, and therapeutic response patterns [114].

In osteosarcoma, PDO-based chemosensitivity testing has demonstrated the ability to predict neoadjuvant chemotherapy response and correlate with long-term survival outcomes, supporting the use of organoids as functional biomarkers for individualized therapeutic selection [115]. Within a precision oncology framework, PDOs may therefore provide ex vivo validation of treatment sensitivity beyond molecular profiling alone.

However, organoid systems remain limited by variability in establishment success, incomplete immune and stromal representation, and lack of cross-center standardization [116].

PDX models provide additional in vivo biological fidelity by preserving tumor-microenvironment interactions [117]. In osteosarcoma, PDOX studies have identified potentially effective regimens for drug-resistant disease, including regorafenib- and irinotecan-based strategies, demonstrating that functional modeling may reveal therapeutic vulnerabilities not fully predicted by genomic profiling [118]. Nevertheless, broader clinical implementation remains limited by high cost, technical complexity, prolonged establishment times, and limited immune representation [119].

##### Emerging biofabrication platforms

Emerging biofabrication technologies, including 3D bioprinting and microfluidic tumor-on-chip systems, aim to recreate physiologically relevant tumor architecture and microenvironmental conditions in bone sarcomas [120, 121]. In parallel, 3D printing technologies are increasingly being explored for patient-specific surgical planning, reconstruction, and customized implant fabrication in complex bone sarcoma resections. Recent studies suggest that patient-specific 3D-printed models and guides may facilitate limb-salvage surgery, accurate tumor resection, and reconstruction in musculoskeletal oncology [91].

These platforms may improve the evaluation of therapeutic response, tumor heterogeneity, and metastatic behavior under dynamic experimental conditions. However, despite their translational potential, such systems remain largely preclinical and require further technical standardization and clinical validation before integration into routine precision oncology workflows [122].

#### Liquid biopsy

Liquid biopsy has emerged as a promising extension of precision medicine in primary bone sarcomas by enabling minimally invasive and longitudinal monitoring of tumor-derived biomarkers [123]. Unlike conventional tissue biopsy, which captures only a single anatomical region at one time point, liquid biopsy may better reflect tumor heterogeneity, clonal evolution, and treatment-related molecular changes [123, 124]. Circulating tumor DNA (ctDNA), circulating tumor RNA (ctRNA), circulating tumor cells (CTCs), and extracellular vesicles collectively provide dynamic insight into tumor biology and therapeutic response [125].

Among circulating analytes, ctDNA represents the most extensively investigated biomarker in bone sarcomas. In Ewing sarcoma, tumor-specific EWSR1–ETS fusion sequences enable highly sensitive plasma-based molecular monitoring, with ctDNA levels correlating with tumor burden, treatment response, and clinical outcomes [126, 127]. Persistence of detectable ctDNA following induction chemotherapy has additionally been associated with inferior survival outcomes, supporting potential applications in minimal residual disease (MRD) assessment and early relapse prediction [128]. These findings suggest that fusion-driven sarcomas may be particularly suitable for molecular monitoring approaches.

In contrast, liquid biopsy in osteosarcoma remains more challenging because of extensive genomic instability and the absence of recurrent driver alterations [129, 130]. Nevertheless, dynamic ctDNA changes may still provide clinically relevant information regarding treatment response and impending relapse, potentially offering a broader representation of tumor heterogeneity than localized tissue sampling alone [128].

The clinical role of liquid biopsy in chondrosarcoma remains less established. Although IDH1 and IDH2 mutations are recurrent in some subtypes, biomarker detectability and translational applicability appear more variable than in Ewing sarcoma and osteosarcoma [131].

Additional circulating biomarkers, including ctRNA, CTCs, and extracellular vesicles, may further contribute to molecular monitoring and characterization of metastatic behavior [132–136]. Nevertheless, detection platforms remain technically heterogeneous, and prospective validation in bone sarcoma cohorts is limited [137, 138].

Despite promising early findings, liquid biopsy in bone sarcomas remains an emerging field requiring larger prospective studies and assay standardization. Nevertheless, as precision oncology strategies evolve, liquid biopsy may serve as a valuable complement to tissue profiling, imaging, and functional modeling platforms by enabling real-time molecular monitoring and adaptive therapeutic stratification [123, 139]. The currently investigated liquid biopsy biomarkers, their principal clinical applications, and major limitations in bone sarcomas are summarized in Table 4.

**Table 4:** Liquid biopsy biomarkers and clinical applications in bone sarcomas.

**ctDNA (fusion-based)**	**Ewing sarcoma**	**MRD detection, relapse prediction**	**Emerging–strong**	**Limited large validation**
ctDNA (genomic instability)	Osteosarcoma	Response monitoring	Emerging	Variable sensitivity
ctRNA	Ewing sarcoma	Functional activity tracking	Early	RNA instability
CTCs	Metastatic disease	Biological characterization	Early	Low detection rate
Exosomal markers	Investigational	Microenvironment insight	Early	Standardization lacking

ctDNA: circulating tumor DNA; ctRNA: circulating tumor RNA; CTCs: circulating tumor cells; MRD: minimal residual disease.

## Conclusions

Despite rapid advances in genomic profiling and experimental modeling, the clinical translation of precision medicine in primary bone sarcomas remains incomplete [140]. While molecular characterization has refined diagnostic classification and improved biological understanding, its integration into routine therapeutic decision-making remains limited.

A major barrier is the rarity of bone sarcomas, which restricts cohort sizes and complicates the design of biomarker-driven prospective trials [141, 142]. Most genomic, liquid biopsy, and functional modeling studies are derived from relatively small or retrospective cohorts, limiting statistical power and generalizability. In addition, the pronounced inter- and intratumoral heterogeneity observed in these malignancies, particularly in genomically unstable osteosarcoma, reduces the predictive value of single-time-point molecular assessments and contributes to therapeutic resistance [143].

Methodological variability further constrains clinical implementation. Differences in sequencing platforms, bioinformatic pipelines, ctDNA detection thresholds, and organoid establishment protocols hinder standardization and cross-study comparability. Moreover, certain precision platforms, such as PDX models, require timelines that may not align with the urgency of clinical decision-making in aggressive disease settings.

Future progress will likely depend on integrated, multi-layered frameworks rather than isolated modalities. Genomic stratification can define biological subtypes and candidate therapeutic targets; functional modeling platforms may validate drug sensitivity in patient-specific systems; and liquid biopsy approaches can enable longitudinal monitoring of clonal evolution and MRD. The coordinated application of these strategies, supported by multi-institutional collaboration and biomarker-driven clinical trials, represents a promising pathway toward clinically actionable precision oncology in bone sarcomas [142].

Ultimately, precision medicine in bone sarcomas should not be viewed as the pursuit of a single actionable mutation, but rather as an adaptive framework integrating molecular diagnostics, therapeutic targeting, and dynamic disease monitoring. Such an approach acknowledges the profound biological complexity of these malignancies and provides a pathway toward more individualized and biologically informed patient care.

## Abbreviations

3D: three-dimensional
CAR-T: chimeric antigen receptor T-cell
CTCs: circulating tumor cells
ctDNA: circulating tumor DNA
ctRNA: circulating tumor RNA
EWSR1–ETS: EWS RNA-binding protein 1–ETS transcription factor fusion family
IDH1/2: isocitrate dehydrogenase 1 and 2
IoT: Internet of Things
MRD: minimal residual disease
PARP: poly(ADP-ribose) polymerase
PDOs: patient-derived organoids
PDX: patient-derived xenograft
TCR-T: T-cell receptor-engineered therapies
TKIs: tyrosine kinase inhibitors

## Declarations

### Copyright

© The Author(s) 2026.
